# Digital smile design-driven minimally invasive lithium disilicate laminate veneers in the aesthetic zone: a 6-year follow-up case report

**DOI:** 10.3389/fdmed.2026.1746318

**Published:** 2026-02-17

**Authors:** Anh Tuan Dang, Anh Phuong Thi Nguyen, Ly Thuy Thi Le, Hai Thanh Pham, Rim Bourgi

**Affiliations:** 1 Department of Prosthodontics, Faculty of Dentistry, Haiphong University of Medicine and Pharmacy, Haiphong, Vietnam; 2 Department of Odonto-Stomatology, Haiphong Medical University Hospital, Haiphong, Vietnam; 3 Department of Oral Surgery and Periodontics, Faculty of Dentistry, Haiphong University of Medicine and Pharmacy, Haiphong, Vietnam; 4 Department of Biomaterials and Bioengineering, INSERM UMR_S 1121, University of Strasbourg, Strasbourg, France; 5 Department of Restorative Sciences, Faculty of Dentistry, Beirut Arab University, Beirut, Lebanon; 6 Department of Restorative Dentistry, School of Dentistry, Saint-Joseph University, Beirut, Lebanon

**Keywords:** adhesive dentistry, adhesive protocol, aesthetic dentistry, digital planning, digital smile design, laminate veneer, lithium disilicate, long-term follow-up

## Abstract

The foundational principle of minimally invasive dentistry is the preservation of sound tooth structure. Its efficacy is directly dependent upon strict adherence to an optimized clinical protocol and mastery of dedicated instrumentation. This case report illustrates a conservative Digital Smile Design (DSD) - guided approach to ceramic laminate veneer rehabilitation of the maxillary anterior dentition, significantly enhancing the patient's smile. A 29-year-old female presented with the chief complaint of an unattractive smile due to an imbalanced smile line, tooth form, tooth angle and color in the aesthetic zone. DSD concepts were carried out using the Photoshop software, followed by diagnostic wax-up and mock-up to reconstruct aesthetically harmonious tooth form and color. The patient approved the minimally invasive treatment with a gingivectomy using a scalpel and veneers for 10 anterior teeth instead of the orthodontic treatment. A temporary prosthesis based on a silicone index was used to aid the conservative tooth preparations and lithium disilicate (LDS) veneers were bonded under total isolation conditions with a rubber dam. The adhesive protocol employed a total-etch technique, followed by the application of a bonding agent, intermediate dentin sealing (IDS) and a light-cured adhesive resin to optimize bonding efficiency. Following proper planning and sequencing, the outcomes successfully achieved fulfilled the patient's aesthetic demands and were reevaluated as well-maintained using the Modified United States Public Health Service (USPHS) criteria at 6-years follow-up. The application of DSD followed by LDS veneer restorations allows a minimally invasive approach with an excellent long-term outcome for the disharmonious anterior teeth, especially in high-aesthetic-demand patients.

## 1 Introduction

The perception of aesthetic appearance is a significant determinant of psychological and social well-being, with societal biases often favoring individuals perceived as attractive, correlating with greater observed success (1, 2). Concurrently, the field of aesthetic dentistry has experienced substantial growth, a trend increasingly propelled by the pervasive influence of social media platforms and digital applications (3). Achieving optimal outcomes for ceramic restoration in the aesthetic zone requires precise planning and execution. Digital Smile Design (DSD) has revolutionized restorative dentistry by providing a comprehensive digital approach. Utilizing specialized software and imaging, DSD enables clinicians to analyze facial and dental proportions and digitally design a harmonious, patient-specific smile before treatment (4, 5). Integrating DSD into the workflow permits clinicians to overcome limitations of subjective assessment, enhances dentist-patient communication by enabling patients to visualize proposed outcomes, fosters collaboration within interdisciplinary teams and increases patient satisfaction and involvement in decision-making (6, 7). Furthermore, DSD serves as a powerful tool that ensures appropriate communication between clinicians and dental laboratories for accurate restoration fabrication (8).

Diagnostic wax-up constitutes a fundamental diagnostic and treatment planning tool for patients being considered for prosthetic restorations. It enables the critical assessment of discrepancies between the existing dentition and the proposed ideal morphology, including evaluations of tooth size discrepancies, available restorative space, the planned occlusal scheme and potential treatment requirements within the opposing arch (9). Furthermore, this allows for clinical evaluation of the provisional restorations and provides an opportunity for the patient to request modifications at an early stage of treatment planning. In terms of diagnostic role, it provides the basis for fabricating both diagnostic guides and preparation reduction guides (10). The definitive tooth preparation can be precisely executed using an interim prosthesis stabilized intraorally via a putty silicone index and a silicone reduction guide derived from the wax-up. Specifically, reduction guides fabricated from the diagnostic wax-up utilizing either clear or putty matrices permit the clinician to quantitatively assess the adequacy of interproximal, incisal and facial reduction for each tooth during preparation, which minimizes enamel removal (11). The pursuit of minimally invasive aesthetic restorations has driven the widespread adoption of ceramic laminate veneers as a primary solution in modern dentistry (12). Among available ceramic materials, lithium disilicate (LDS) has emerged as the preferred choice due to its superior mechanical strength, exceptional optical properties and proven long-term clinical reliability (13). Its high flexural strength, ranging between 350 and 400 Megapascal (MPa), allows its use in monolithic restorations, providing a balance between aesthetics and function (14). Compared to traditional feldspathic ceramics, LDS offer an optimal balance of high fracture resistance and natural translucency, making them particularly suitable for aesthetic rehabilitation of anterior teeth as well as adoptable in minimally invasive prosthodontic procedures, such as veneers, inlays and onlays (15). Clinical studies consistently demonstrate high survival rates and minimal complication rates for LDS restoration (16–18). Besides, achieving long-term success with these restorations necessitates careful case selection, conservative preparation protocols and precise adhesive cementation techniques (19, 20). Crucially, enamel preservation optimizes bonding outcomes; LDS veneers exhibit superior adhesion to enamel, conferring enhanced longevity and significantly greater survival rates relative to dentin-bonded restorations (21).

On the other hand, the successful rehabilitation of aesthetically disharmonious anterior dentition, particularly in cases presenting with complex biomechanical and periodontal challenges such as altered passive eruption (APE) and Angle Class II division 2 malocclusion which require a meticulously planned, minimally invasive approach. In such clinical scenarios, conventional treatment planning may fall short in addressing the intricate interplay between aesthetic ideals, functional demands and biological preservation. When combined with the high strength, excellent aesthetics, and conservative preparation protocols afforded by the LDS laminate veneers, this digital workflow ensures an optimal balance between maximal tissue preservation and long-term restorative success (22).

This case report illustrates the application of this integrated protocol in a patient with anterior aesthetic disharmony, highlighting how DSD-driven planning and LDS veneers can effectively address both aesthetic deficiencies and underlying structural challenges through a minimally invasive intervention.

## 2 Case presentation

### 2.1 Timeline

A summary of key treatment and follow-up events is provided in Table 1.

**Table 1:** Timeline of the treatment and follow-up.

Time points	Clinical procedure
Initial Visit	Report of chief complaint, clinical/radiographic examination, diagnosis.
1st appointment	DSD analysis and planning, diagnostic impressions.
2nd appointment	Diagnostic wax-up fabrication, mock-up, try-in.
3rd appointment	Gingivectomy (#11, #12), final tooth preparation.
4th appointment	Final impression, laboratory fabrication of LDS veneers.
5th appointment	Try-in and definitive adhesive cementation.
2-Year Follow-up	Clinical evaluation, composite repair of lingual cusp fracture on #15.
6-Year Follow-up	Comprehensive clinical evaluation, USPHS scoring, periodontal assessment.

### 2.2 Clinical and radiologic evaluation

**Figure 1:**
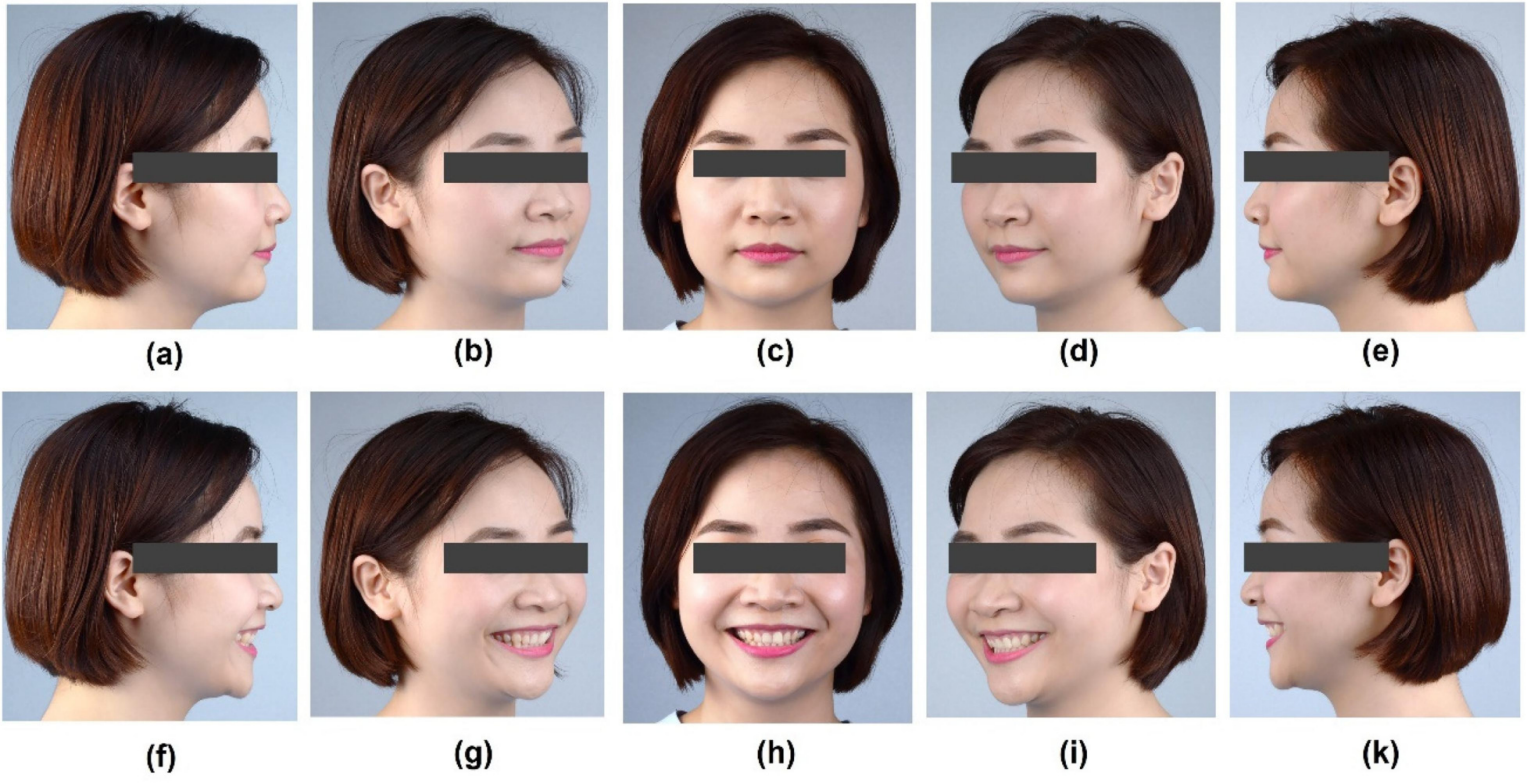
Portrait photographs of the patient's initial situation. **(a)** 90 degree right at rest, **(b)** 45 degree right at rest, **(c)** frontal view at rest, **(d)** 45 degree at rest, **(e)** 90 degree at rest, **(f)** 90 degree right smile, **(g)** 45 degree right smile, **(h)** frontal view smile, **(i)** 45 degree left smile, **(k)** 90 degree left smile.

A 29-year-old female patient presented to the clinic with the chief goal of improving her smile, becoming “brighter” and “more attractive” than currently (Figure 1). The written consent for each of the following procedures, including the publication of photos of the steps of the treatment, was signed. The Declaration of Helsinki and the PROCESS checklist were fully implemented in the present study (23).

**Figure 2:**
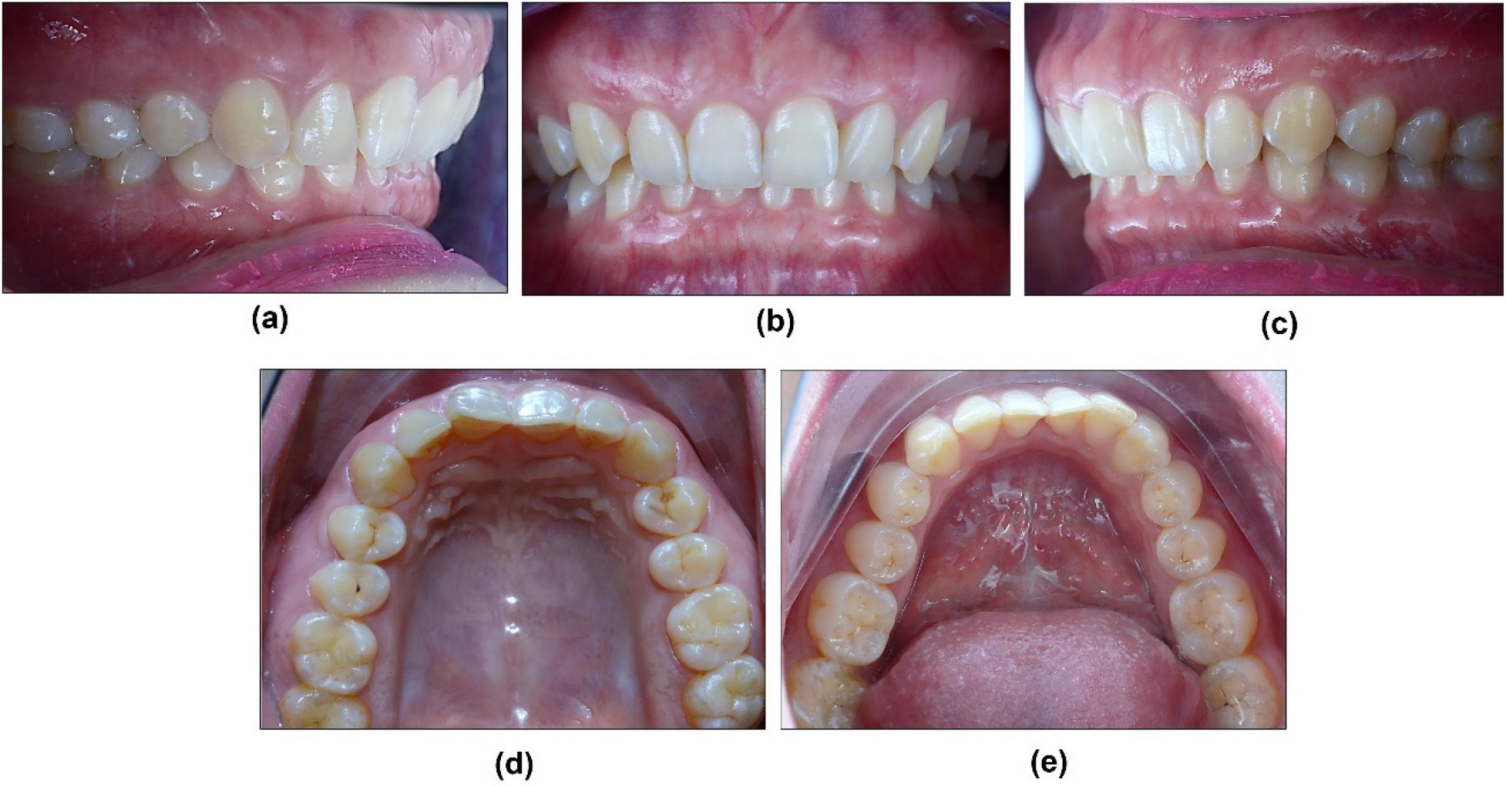
Intra-oral picture of the patient's initial situation. **(a)** Right side view, **(b)** Frontal view, **(c)** Left side view, **(d)** Maxillary occlusal view, and **(e)** Mandibular occlusal view.

The patient's oral hygiene was well-maintained, classified as excellent according to the Simplified Oral Hygiene Index (24). The smile of the patient exposed to the second maxillary premolar. She was diagnosed with good periodontal health: periodontal probing depths (PPD) were within 1–2 mm, without bleeding on probing (BOP). Notably, her #11 and #12 teeth present Altered Passive Eruption (APE) type 1A, which showed normal in width but reduced 1.0–1.5 mm in length, making the teeth a short and asymmetrical size, with square-looking clinical crowns. Her incisors presented a proximal rotation labial combination with a palatal inclining axis of the clinical crown, which resulted in reduced upper lip support. Furthermore, occlusal examination reveals a mild Angle Class II subdivision 2 malocclusion, characterized by the bilateral first molars and canine relationship. Clinical analysis of the anterior dentition indicated a normal horizontal overlap (overjet). Vertically, a mild deep overbite was observed, concomitant with a lingual inclination of the maxillary incisors (Figure 2).

**Figure 3:**
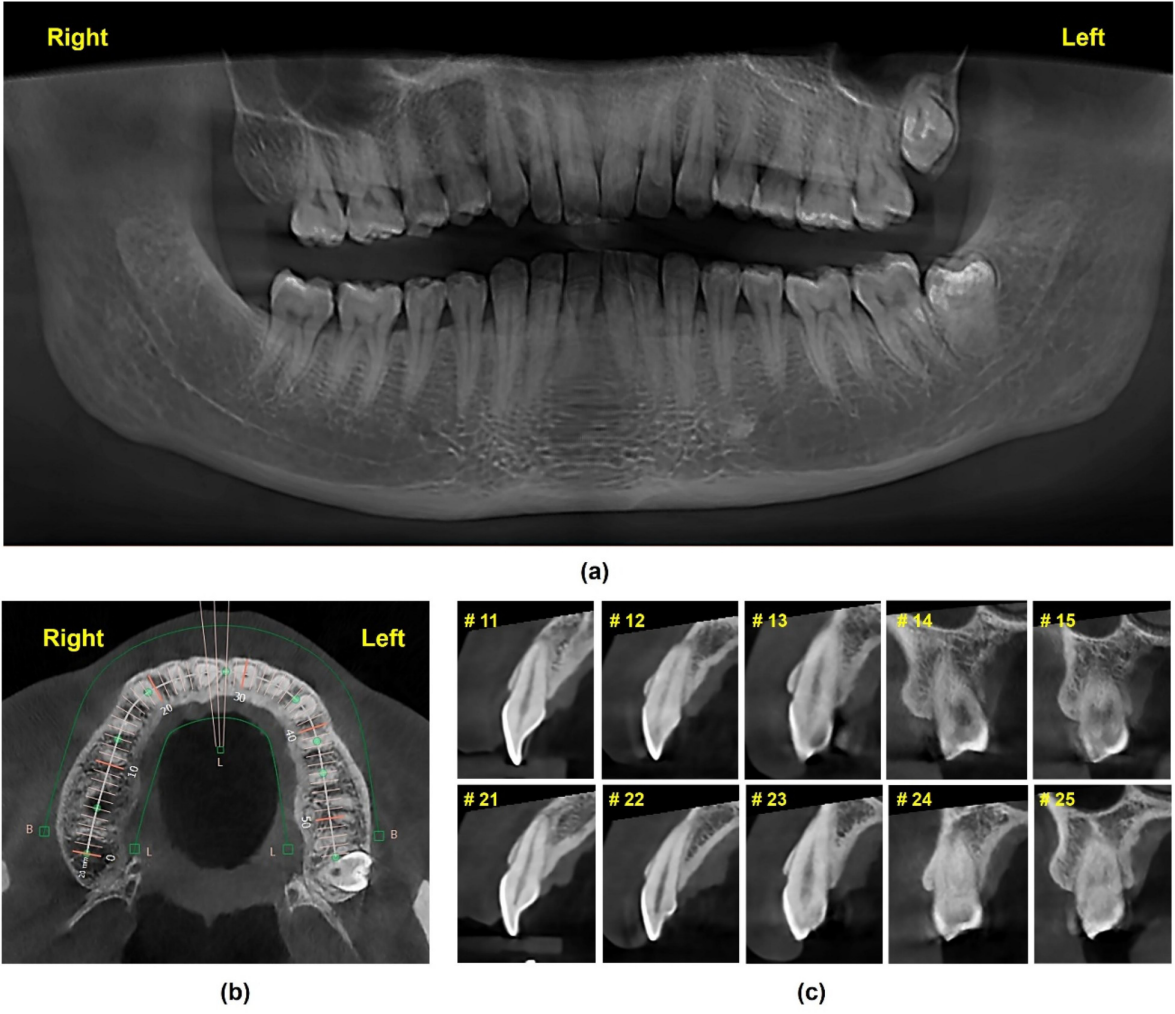
Initial radiographic data of the patient. **(a)** Panoramic radiograph, **(b)**, **(c)** Sagittal sections of teeth #15 to #25 at Multiplanar Reformat Mode (MPR) of the Conebeam CT (CBCT) radiograph.

Dental history including third molar extraction of #18 and #48. The radiologic status of the remaining teeth was further evaluated using panoramic and Cone-beam computed tomography (CBCT) radiographs (Dentri-S Model, HDX Will Group, Korea) and CBCT analysis software (Ez3D-I, Vatech, Korea) (Figure 3).

### 2.3 Diagnostic and differential treatment planning

The aesthetic diagnosis was based on DSD analysis of standardized photographs, identifying discrepancies in the gingival zeniths, incisal edge positions, and tooth proportions. The occlusal diagnosis of Angle Class II division 2 malocclusion indicated that functional loads would be concentrated on the incisal edges of the central incisors and palatal aspects of the canines (11). The presence of APE Type 1A on #11 and #12 necessitated crown lengthening to achieve ideal crown proportions. Orthodontic treatment combined with bleaching: This option would address the malocclusion and tooth inclination biologically, ideal for long-term health. However, the patient declined due to the duration of treatment and concerns about appliance visibility. Combined orthodontic-prosthetic therapy: A segmented approach to correct major discrepancies followed by veneers. This was considered more complex and time-consuming. Minimally invasive prosthetic rehabilitation with DSD-guided LDS veneers: This plan was selected based on patient preference for a shorter treatment time, the predictability offered by the DSD mock-up for visualizing the outcome, and the ability of LDS veneers to provide sufficient strength and aesthetics with minimal tooth preparation. The plan included gingivectomy on #11 and #12 to correct APE.

### 2.4 Treatment planning using the DSD concept

**Figure 4:**
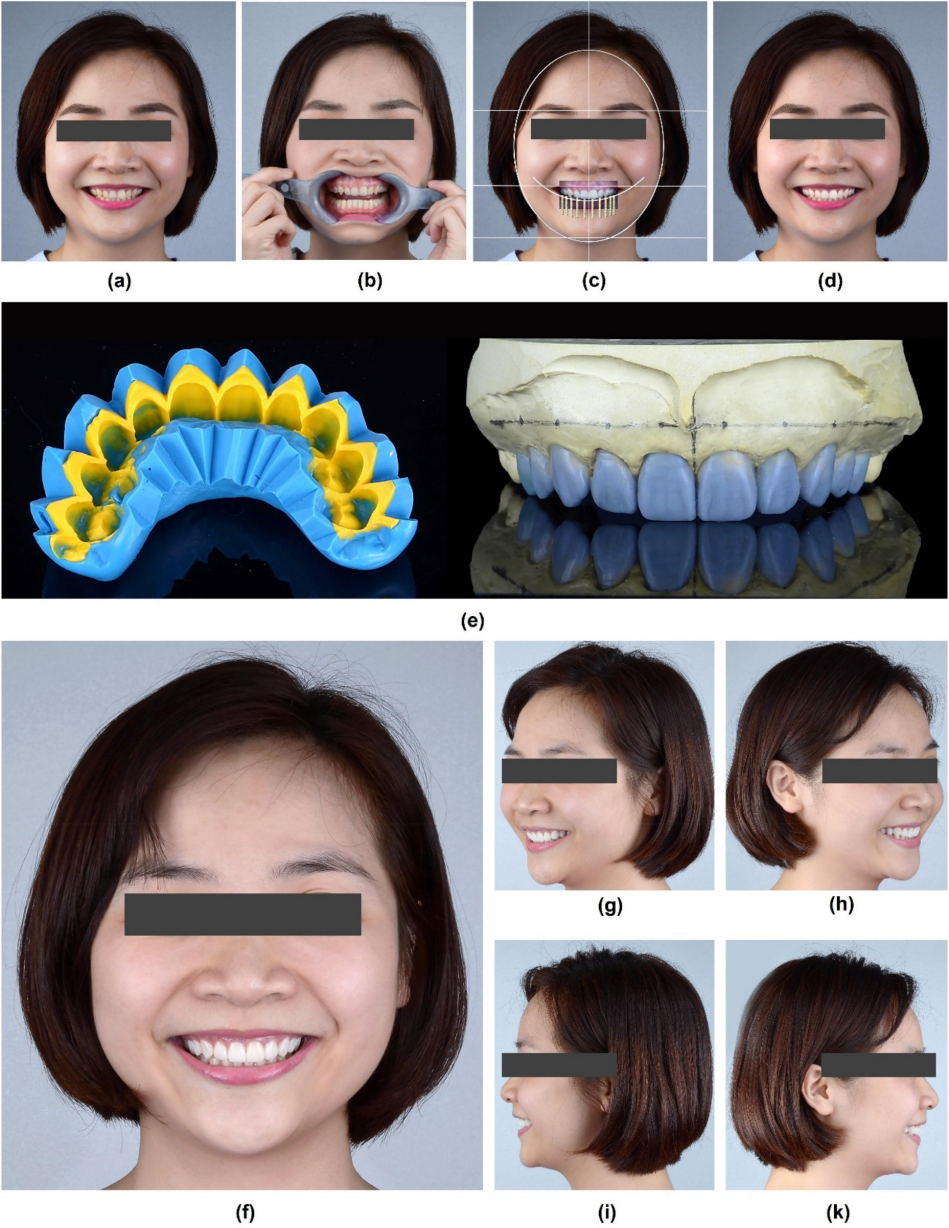
DSD procedure. **(a)** Initial frontal smile picture, **(b)** Frontal smile with retractor, **(c)** DSD planning using the superimposition of intra-oral frontal smile and frontal smile pictures with guided lines and rulers for the measurement and design of future clinical crown height and width generated by the Photoshop software, **(d)** Stimulation of the future smile, **(e)** DSD-driven diagnostic wax-up and fabrication of the silicone index, **(f)** Frontal smile with mock-up, **(g)** 45 degree right site view of smile with mock-up, **(h)** 45 degree left site view of smile with mock-up, **(i)** 90 degree right site view of smile with mock-up, and **(k)** 90 degree left site view of smile with mock-up.

To create a detailed treatment plan, the clinician collected a full set of the patient's portrait photos along with intra-oral images. DSD analysis was performed using Photoshop software (Adobe Photoshop CC 2015, USA), which established guidelines superimposing reference lines to digitally design a new smile harmony. This digital design served as a blueprint for the diagnostic wax-up (Figures 4a–d).

### 2.5 Diagnostic wax-up and mock-up

The patient was informed of the need for a diagnostic mock-up to assess restorative options. Firstly, the impressions of both arches were made with PVS material (Honigum putty and light, DMG, Germany) and poured out twice with type IV stone (Fujirock, GC, USA). The maxillary occlusal plan was recorded using a facebow (Elite Facebow, model: BIOAACP0452, Bioart, Brazil) and mounted in a semi-adjustable articulator (Bioart Articulator A7, Bioart, Brazil). Then, two diagnostic wax-ups (Gray Wax GEO Classic, Renfert, USA) were created by 2 different experienced technicians, which stimulated 2 different tooth forms and surface textures (Figure 4e), following the DSD designated template to achieve an aesthetically pleasing dental arrangement that harmonizes with the patient's facial features.

**Figure 5:**
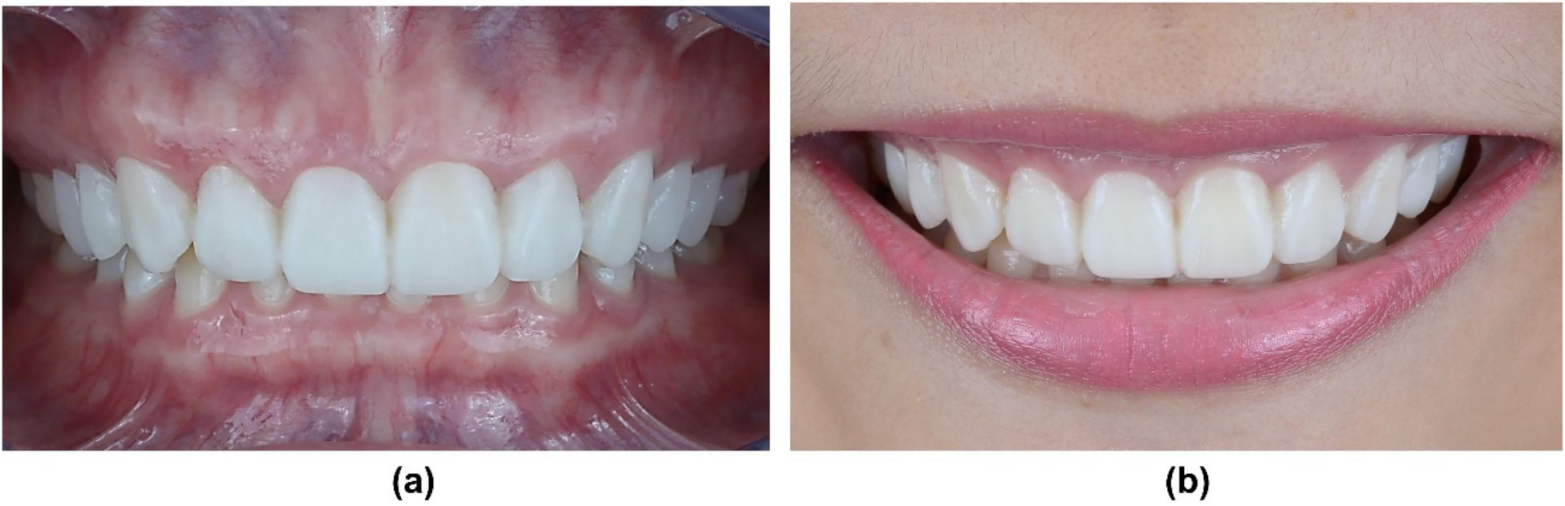
Mock-up. **(a)** Frontal intra-oral and **(b)** Frontal smile views after placement of the mock-up.

Then, the mock-ups were performed in the mouth using temporary resin (Luxatemp Star A2, DMG, Germany) with a putty guide (Honigum putty and light, DMG, Germany), and the results were evaluated by the clinician and patient (Figures 4f–i,k and Figure 5).

### 2.6 Minimally invasive teeth preparation and laboratory steps

**Figure 6:**
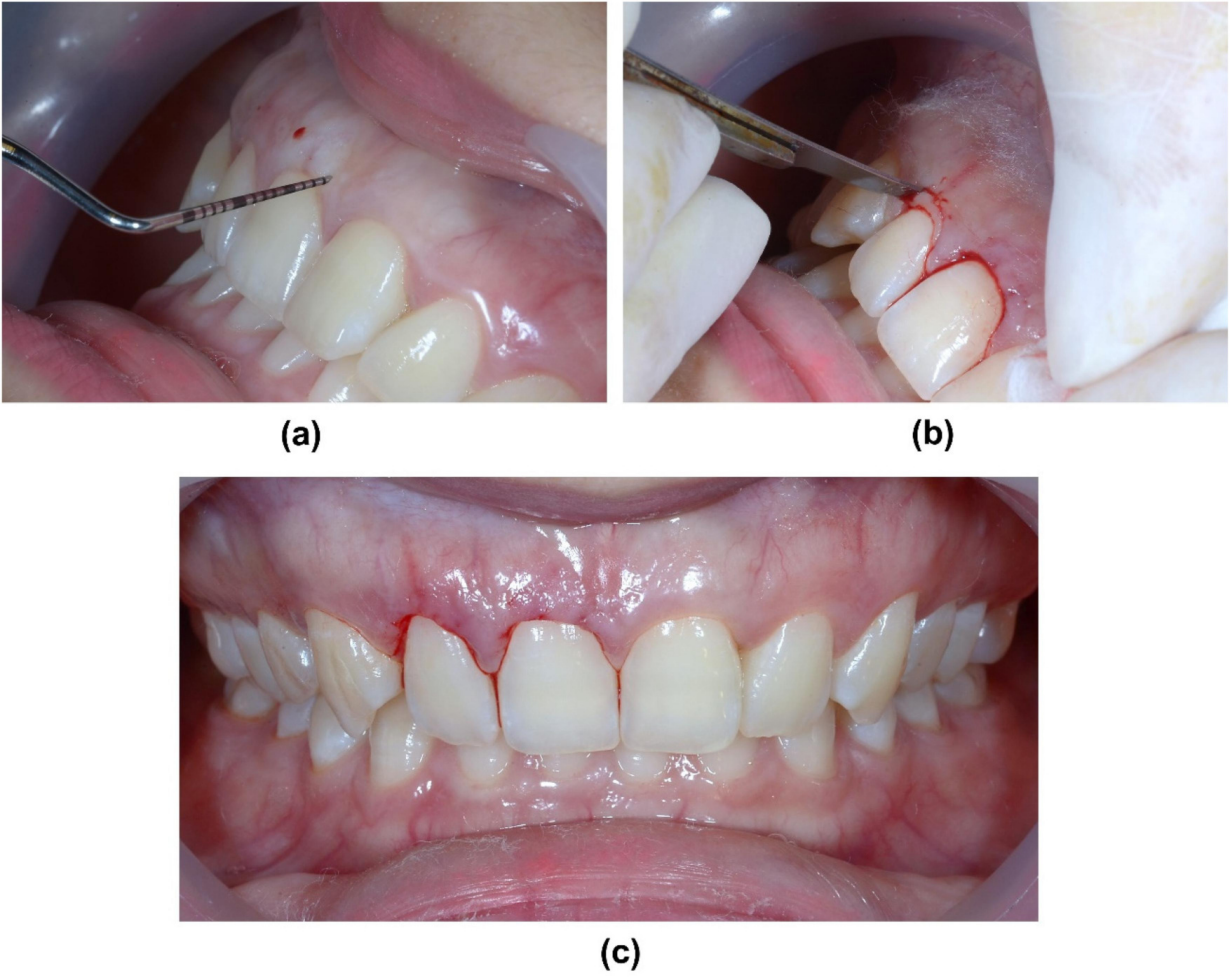
Gingivectomy at #11 and #12. **(a)** Marking the bleeding points following the DSD planning at future Zenith points of incisors by periodontal probe, **(b)** Gingivectomy using no 11 surgical blade, **(c)** Immediate result after gingivectomy.

The treatment plan included crown lengthening by performing a 1.0–1.5 mm gingivectomy at #11 and #12 using a surgical blade (No. 11, Feather Safety Razor Co. Ltd., Osaka, Japan) (Figure 6).

**Figure 7:**
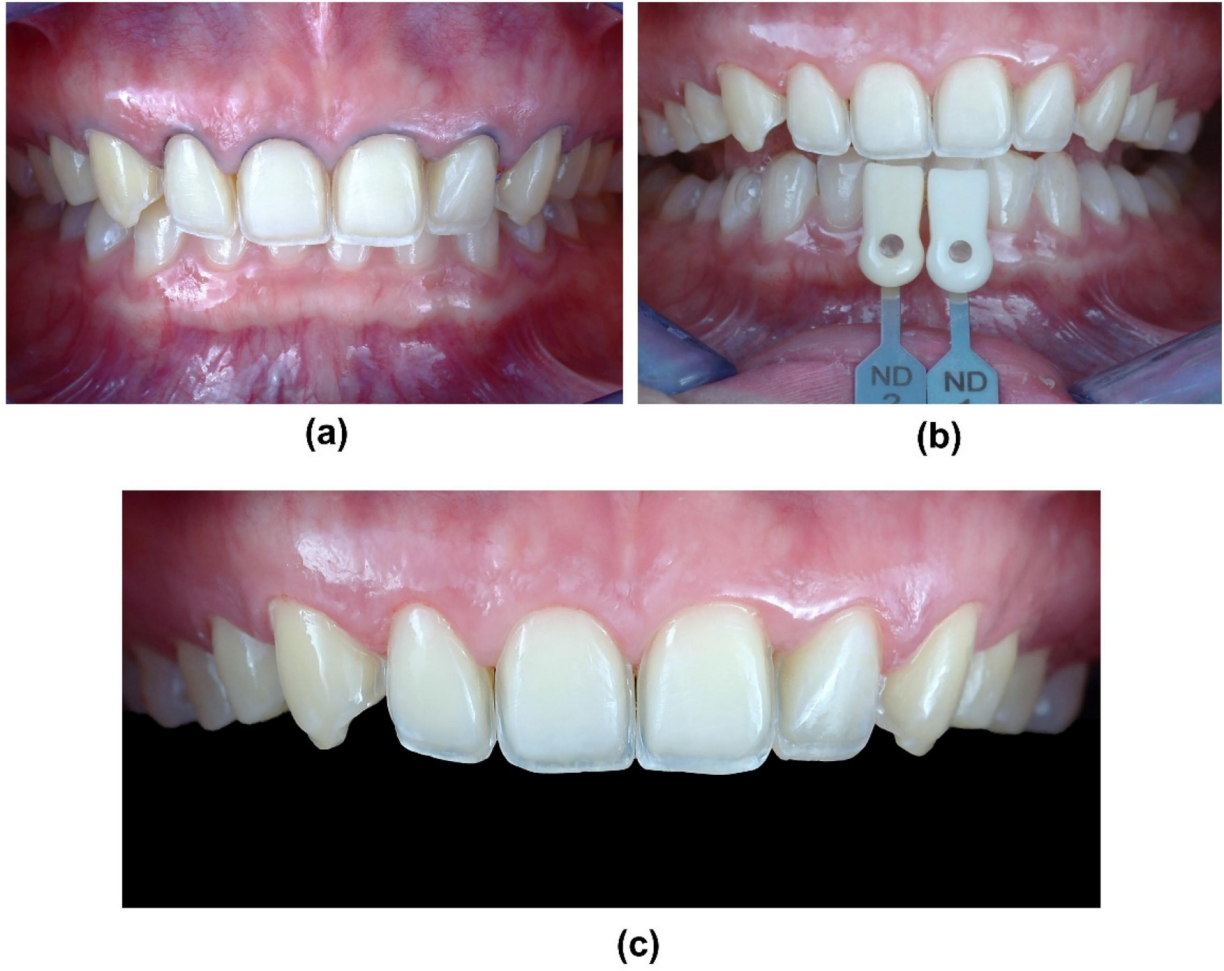
Minimal invasive teeth preparation follows the putty silicone reduction guide. **(a)** Prepared teeth with retraction cords in place, **(b)** Shade matching, **(c)** Frontal view of teeth preparation from tooth #15 to #25.

Subsequently, a diamond bur kit for laminate veneers was used (Ceramic Laminate Veneers Kit, Ref. 9933K3 000, LOT 797593, Komet, Germany) for minimal invasive tooth preparations, with reductions of 0.3–0.5 mm, using a putty silicone reduction guide derived from the diagnostic wax-up. Then, double-cord technique was performed to ensure the prepared tooth margin is exposed for an accurate impression (Cords #00 and #000, Ultrapak, Ultradent Inc., USA). The final tooth preparations were polished using a list of diamond polishing burs and wheels to further create smooth, precise, and clean surfaces for effective bonding of LDS veneers. (Soft Flex, 3M, USA). For the purpose of precise tooth color determination and to standardize communication with the dental laboratory, standardized photographs of the tooth preparations were obtained. The imaging setup consisted of a Digital Single Lens Reflex (DSLR) camera (D7000, Nikon, Japan) paired with a macro lens (Tokina AT-X M100 PRO D, Tokina, Japan). Each photograph incorporated both an A–D shade guide (628587, Ivoclar Vivadent) and an IPS Natural Die Material Shade Guide (ND1-9, Ivoclar Vivadent) as reference standards (Figure 7).

**Figure 8:**
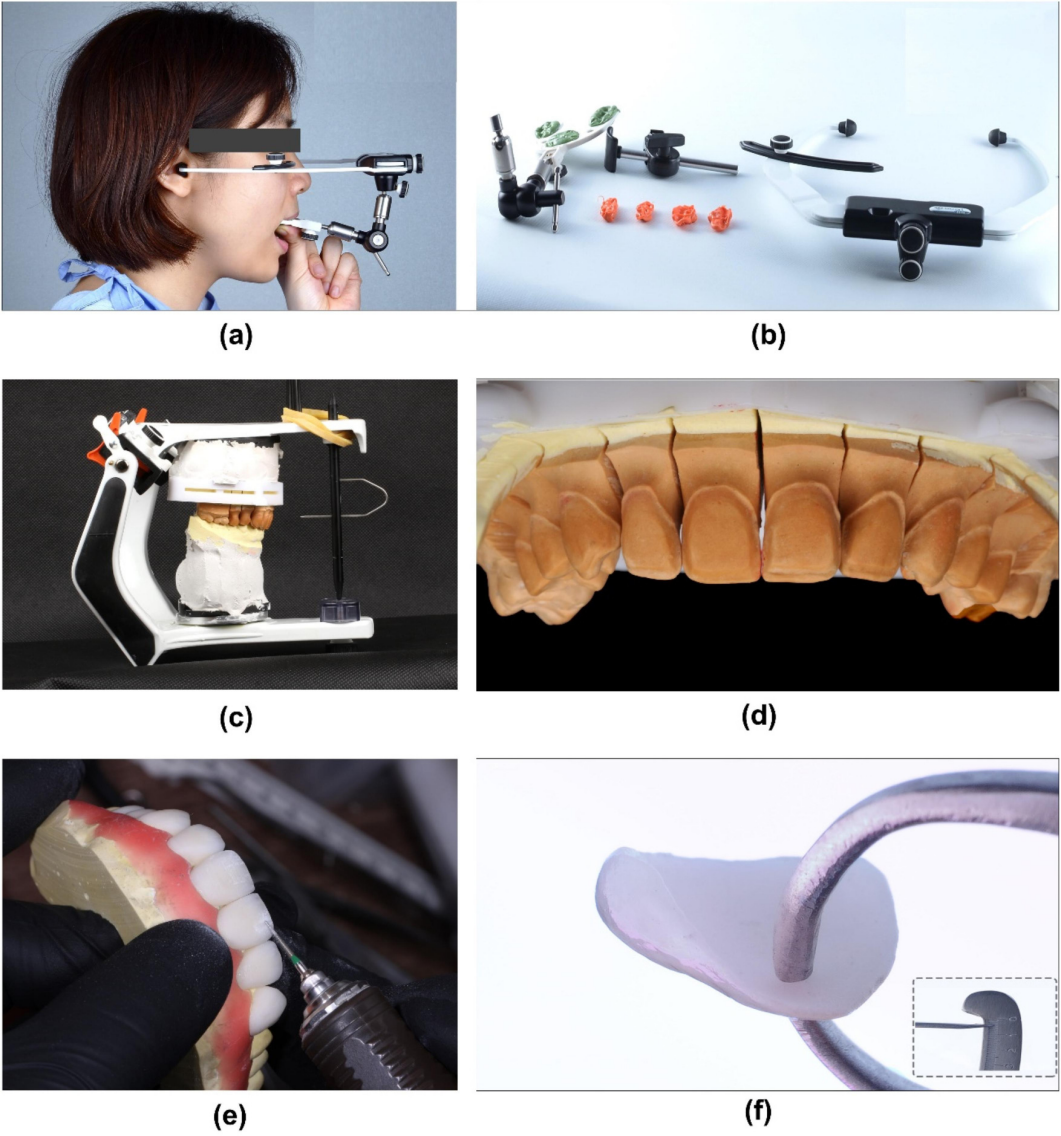
Fabrication of LDS press veneers in the laboratory. **(a)** Record the maxillary position using facebow, **(b)** The facebow and bite registration, **(c)** Working cast and maxillary position was transferred to the semi–adjustable articulator, **(d)** Working cast with dye separation, **(e)** Veneer's texture adjustment, **(f)** Veneer thickness measurement displayed at an average of 0,3–0,5 mm at the coronal part.

The definitive impressions (Honigum Putty and Light, DMG, Germany) of the abutment teeth were poured in type IV dental stone (Fujirock, GC America, USA) to fabricate the master cast. The resultant cast was then remounted on the articulator to accurately simulate the patient's initial occlusal relationships. Then, the porcelain pressed laminate veneers were manufactured (IPS Emax Press HT A2 Ingot, Ivoclar Vivadent, Schaan, Liechtenstein) from the final wax-up and then further adjusted (sculpturing and staining) to create the natural teeth's morphology and surface texture (Figure 8).

### 2.7 Veneers try-in and cementation procedure

**Figure 9:**
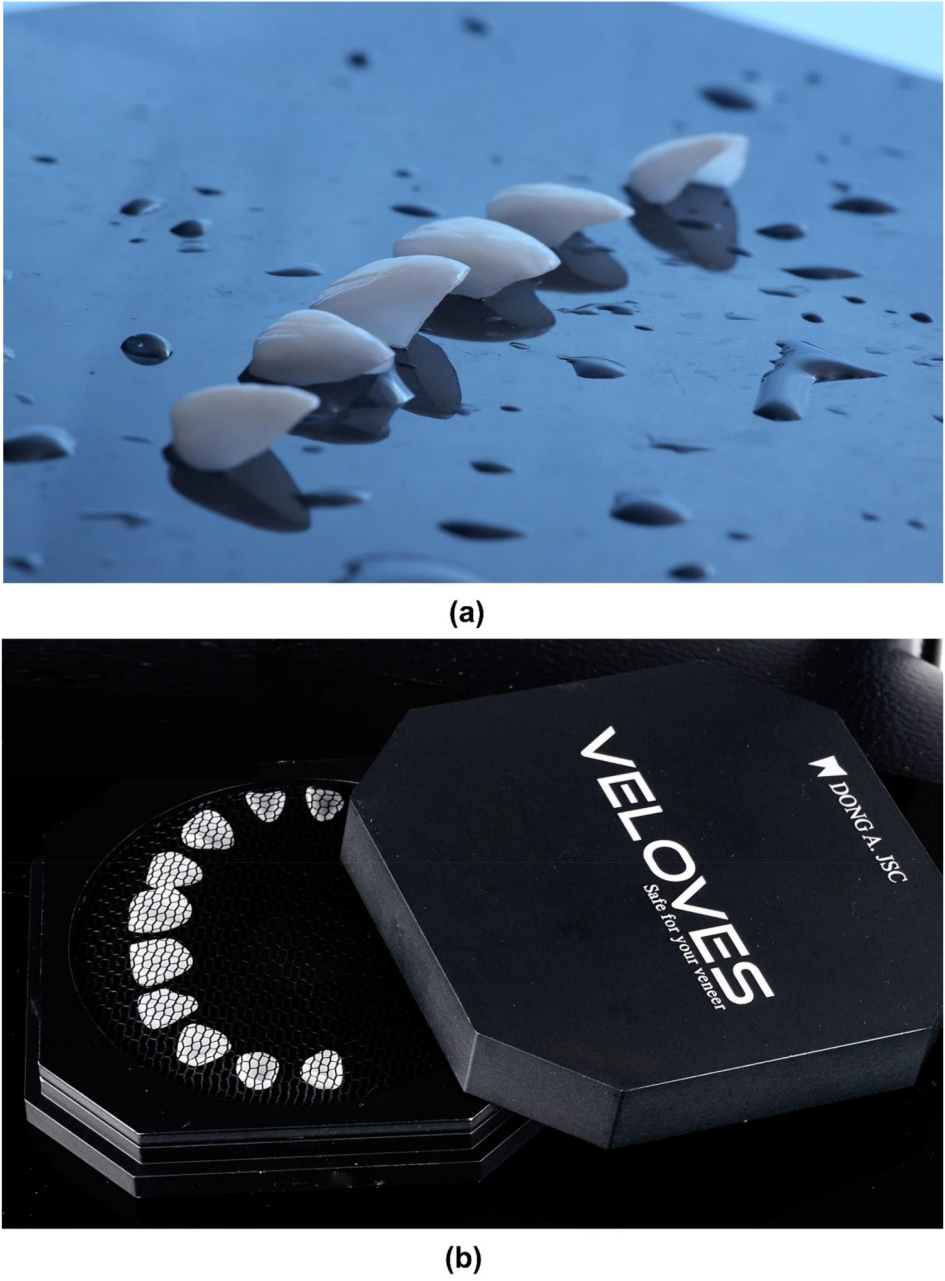
LDS veneers. **(a)** Photograph of veneers, **(b)** LDS veneers were kept in the Pretreatment Patch Box.

The Try-in was done using the try-in paste (Try-in, Neutral, Variolink Aesthetic, Ivoclar Vivadent, Schaan, Liechtenstein) before performing the definitive cementation. LDS veneers were kept in the Pretreatment Patch Box (Veloves, Dong A. JSC, Hanoi, Vietnam) before the treatment process. Then, they were treated first with 5% hydrofluoric acid (IPS Ceramic Etching Gel, Ivoclar Vivadent, Schaan, Liechtenstein) for 20 s, followed by thoroughly rinsing and air-drying. Then, the veneers were cleaned with phosphoric acid (Total Etch, Ivoclar Vivadent, Schaan, Liechtenstein) and a single layer of silanized (Monobond Plus, Ivoclar Vivadent, Schaan, Liechtenstein) for 60 s (Figure 9).

**Figure 10:**
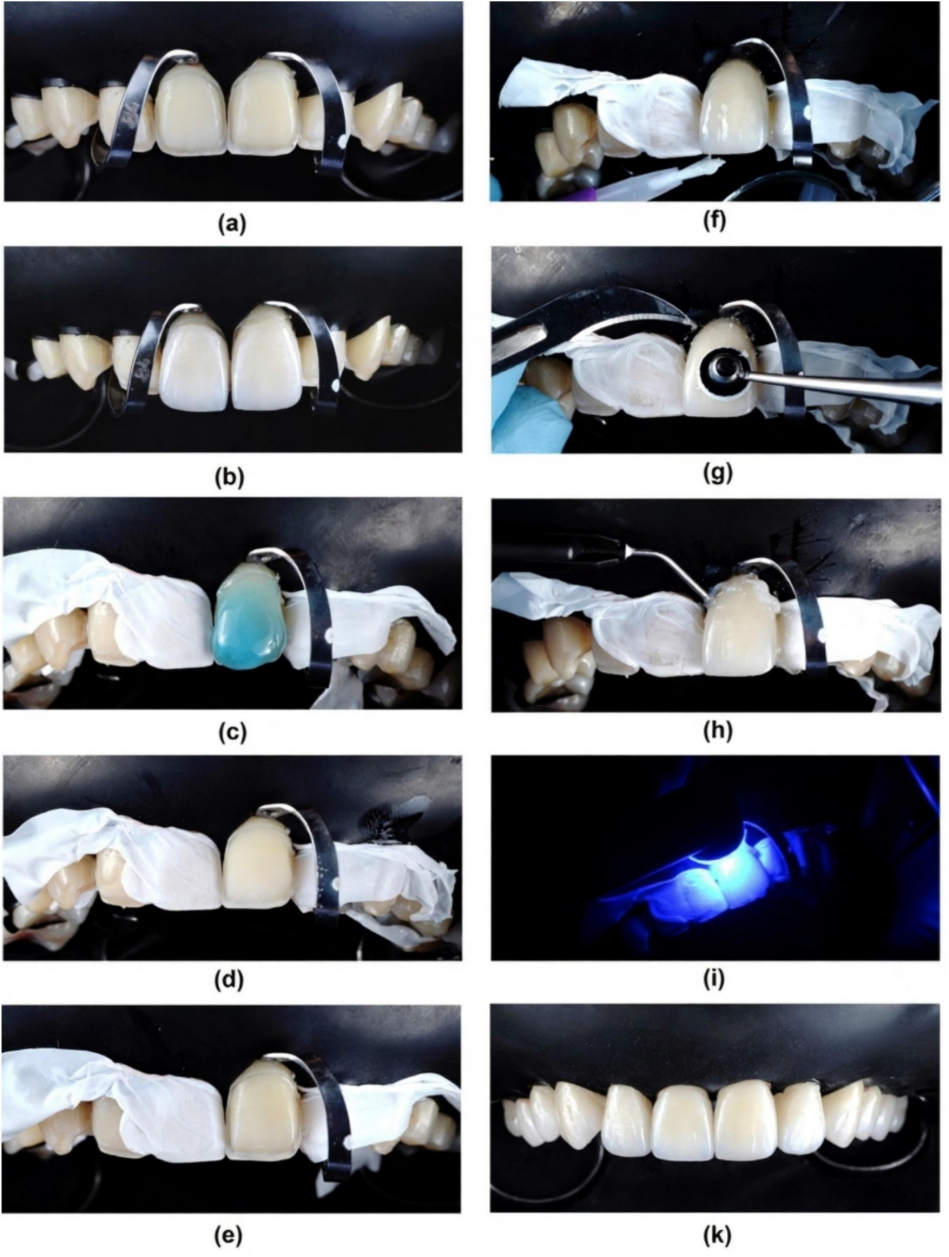
Cementation procedure of LDS veneers. **(a)** Isolation with rubber dam and clamps, **(b)** Try-in, **(c)** Etching enamel surfaces with acid phosphoric 37%, **(d)** Air-dry the tooth surface, **(e)** Apply the bonding agent and performing IDS if necessary, **(f)** Cementation using Esthetic Variolink LC, tack cure, removing the excess cement with brush, **(g)** Further remove the excess cement using the #12 blade, **(h)** Application of glycerine gel, **(i)** Polimerization using a blue light-emitting diode (LED) device, **(k)** Frontal view after finishing the cementation.

Total isolation was provided with a rubber dam (Dental Dam, Sanctuary, USA) covering the maxillary anterior region and retained with clamps (Fiesta, Coltene, USA). Then, the teeth were treated first with 37% phosphoric acid (Total Etch, Ivoclar Vivadent, Schaan, Liechtenstein) for 15 s, followed by a single layer of universal bonding agent application (Tetric N-Bond Viva Pen Universal, Ivoclar Vivadent, Schaan, Liechtenstein), and excess was gently removed with gentle air drying for 20 s. The veneers were then taken out from the pad and charged with resin cement in the intaglio surface (Variolink LC Neutral, Ivoclar Vivadent, Schaan, Liechtenstein) and then positioned with continuous digital pressure into their corresponding tooth. The excess of cement was removed with a brush and dental floss (Oral-B Essential Floss, Oral-B, USA), and each restoration received an initial tack cure within 5 s. Subsequently, a thin layer of glycerin gel (Liquid Strip, Ivoclar Vivadent, Schaan, Liechtenstein) was applied to the interphase and then light-cured again for 60 s on every side of the tooth. The final excesses were removed ustotaling a scalpel blade number 12 or a fine diamond string for cleaning the proximal area, and then all surfaces were repolished with the polishing burs and wheels (Figure 10).

### 2.8 Follow-up and clinical evaluation

**Figure 11:**
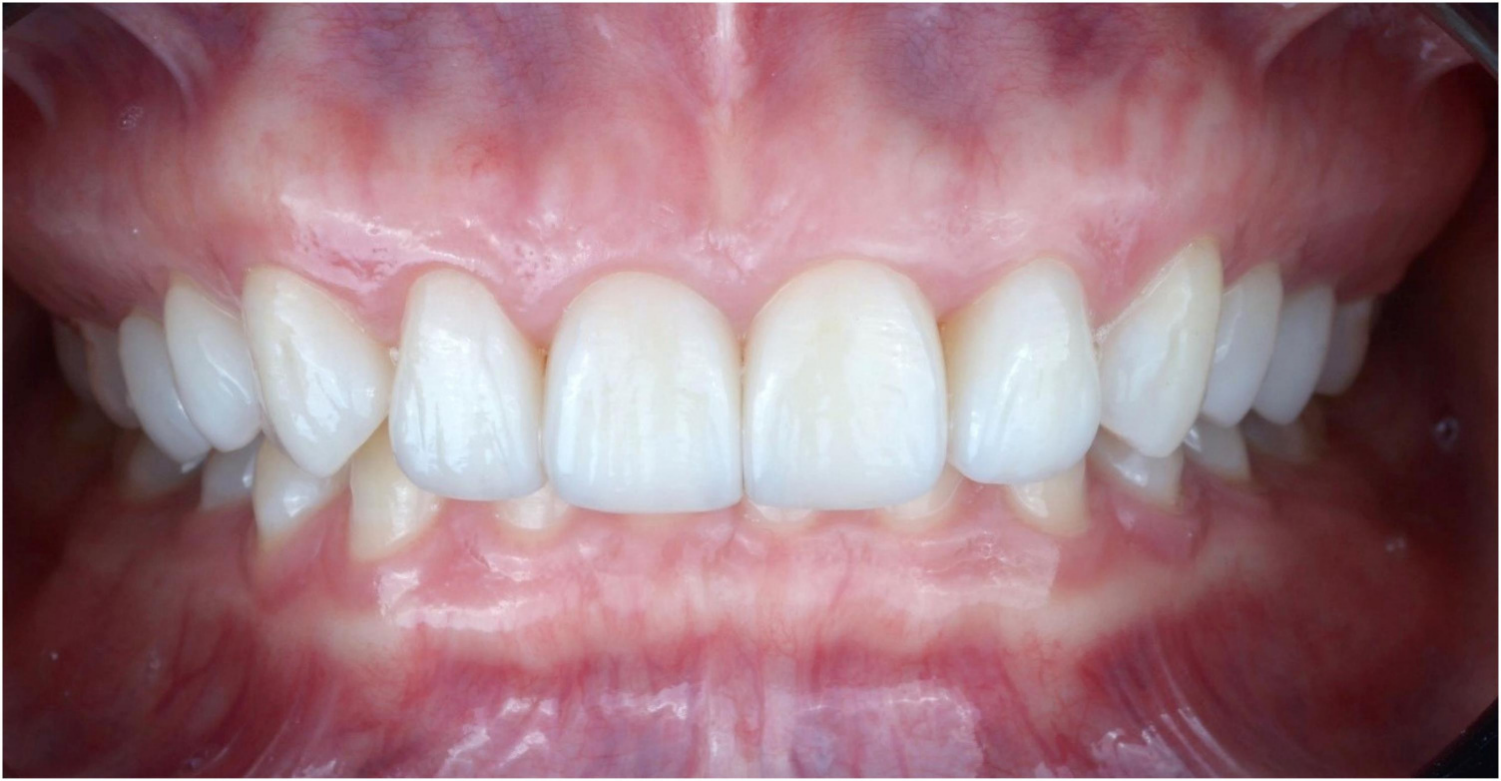
Final LDS veneer restoration (photograph at 1 week post-cementation).

**Figure 12:**
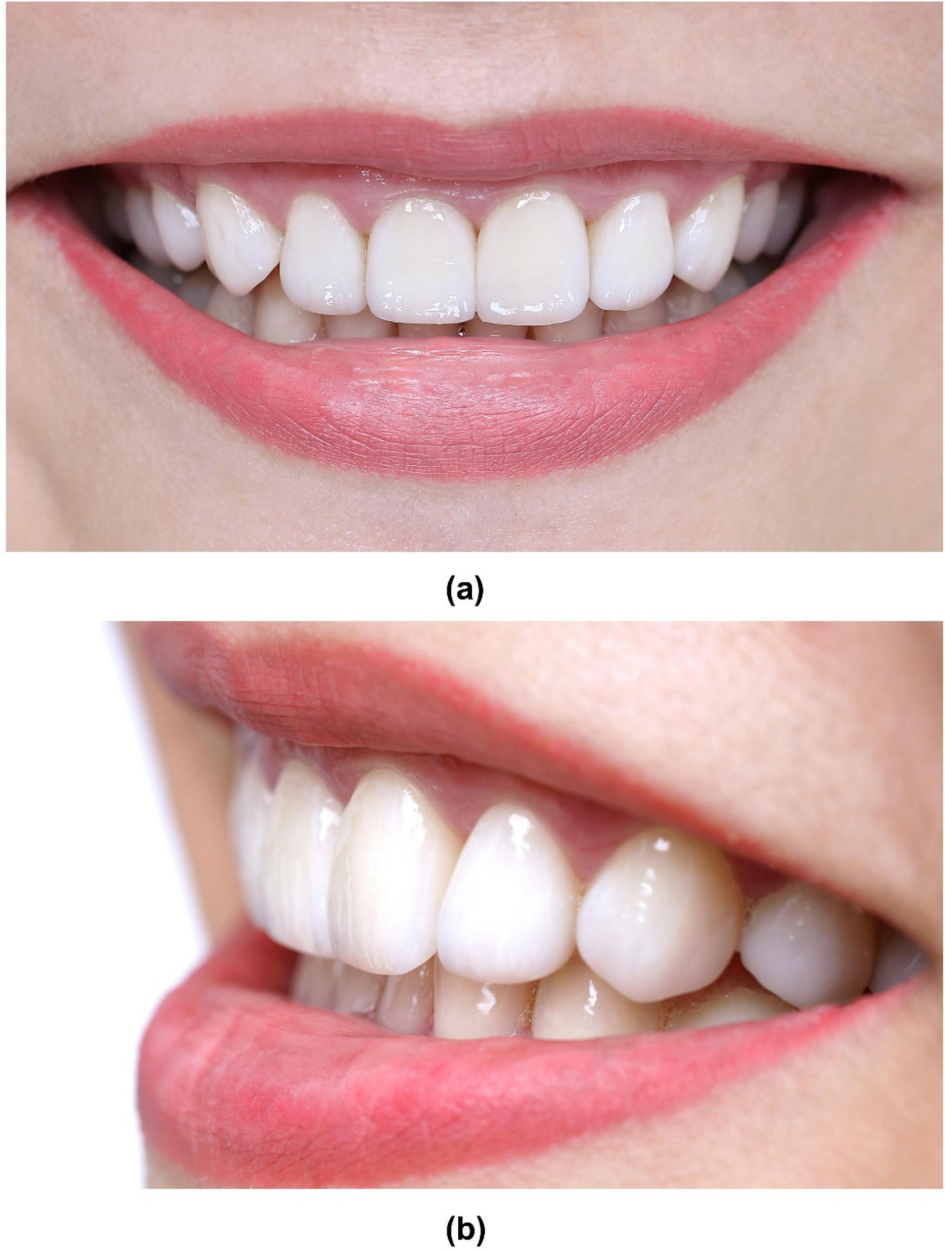
Photographs at 6-year follow-up. **(a)** Frontal smile view, **(b)** Lateral smile view.

The patient was advised on the importance of regular follow-ups on a 6-month recall basis. The restorations were evaluated using the modified USPHS Ryge criteria (Table 2). At the two-year follow-up examination, a lingual cuspal fracture size 1.5 × 2.0 mm (not involving the veneer) was detected on tooth #15. The defective restoration and fracture were restored with a direct composite resin restoration to re-establish the tooth's morphology. Throughout the 6-year observational period, the LDS restorations proved to be clinically successful, providing satisfactory aesthetic and functional results without any incidence of biological or prosthetic-related complications. A slight marginal discrepancy (Bravo score) was noted on two veneers but was considered clinically acceptable and amenable to repolishing. Periodontal health was maintained (PPD: 1–2 mm, no BOP). Occlusal analysis showed stable canine guidance with no signs of parafunction or excessive wear on the veneers (Figures 11, 12).

**Table 2:** Modified USPHS ryge criteria valuation at 6-year follow-up.

Criteria	Score	Description at 6-year recall
Initial visit	Alpha	All veneers present, no debonding.
Retention	Alpha	Excellent match with adjacent natural teeth, no perceptible discoloration.
Color match	Alpha	Excellent match with adjacent natural teeth, no perceptible discoloration.
Marginal adaptation	Bravo	Slight marginal discrepancy or minor discoloration at a few margins; clinically acceptable, repolishable.
Anatomic form	Alpha	Contour and morphology remain ideal, supporting gingival health.
Surface texture	Alpha	Surface remains smooth and glossy, matching adjacent enamel.
Secondary caries	Alpha	No evidence of caries at any restoration margin.

## 3 Discussion

This six-year recall case report demonstrated a 100% survival rate for the LDS veneers. The clinical acceptability of the restorations was longitudinally assessed using a modified USPHS Ryge criteria system. This method involves the direct evaluation of specific qualitative characteristics—including color match, marginal integrity (adaptation and discoloration), anatomical contour, surface texture and evidence of secondary caries. Each criterion is assigned a qualitative score (typically Alpha for ideal, Bravo for clinically acceptable and Charlie for unacceptable) to facilitate standardized monitoring of restoration performance over time (25). In the present case report, the retention, color match, secondary caries, anatomic form and surface texture were evaluated at 0 grade – Alpha; while the marginal adaptation was evaluated at 1 grade - Bravo with a slight deviation from the ideal performance, which possible correction without damage after six years. Several observed slight marginal defects and slight marginal discolorations were not considered definitive failures, as they could be easily repolished or repaired. The results herein, which indicate superior performance of LDS veneers, are corroborated by prior long-term clinical studies reporting high estimated 10-year survival rates of around 95.5% (26–28).

Accurate diagnosis and treatment planning are paramount in aesthetic dentistry, and DSD facilitates this through virtual aesthetic analysis and treatment simulation based on edited photographs or digital patient models (29). This study utilized a dataset comprising standardized extraoral and intraoral photographs of patients. While multiple software solutions exist for digital smile design treatment planning, this study utilizes Adobe Photoshop. This software was prioritized over other available options due to its advanced editing toolkit, which facilitates a non-linear workflow through the use of discrete, editable layers for each design element. This feature, combined with its capacity to export high-resolution images in multiple formats while maintaining exceptional color calibration and sharpness, makes it an optimal tool for precise and reproducible digital wax-ups (30, 31). The reference lines and shapes, as described by Coachman, were applied by using layered analysis to diagnose existing discrepancies and digitally design the proposed prosthetic morphology. A precise superimposition of the patient's frontal facial and intraoral photographs is a critical prerequisite for the accurate transfer of digital design data from the facial to the intraoral context in the Digital Smile Design (DSD) workflow (32). To standardize patient positioning, a pose-fixation headrest and camera viewfinder-integrated alignment guides were employed during image acquisition as previously reported (7). Furthermore, to ensure the stability of the established occlusal plane following the DSD-guided diagnostic mock-up, its orientation was meticulously transferred and verified at each sequential stage of the definitive ceramic restoration fabrication.

The selection of an appropriate preparation design is a critical determinant in the clinical success and longevity of ceramic laminate veneers. Common preparation designs include the featheredge, incisal overlap and window preparations (33). The featheredge design is considered the most conservative, maximizing the preservation of sound tooth structure. However, it may compromise veneer fit and fracture resistance due to minimal incisal reduction and the resulting thin ceramic margin (34). In contrast, the incisal overlap design, which extends the preparation over the incisal edge, enhances restoration stability, improves seating accuracy and provides superior aesthetic outcomes by masking the tooth-veneer interface (35). The window preparation, while also conservative, may offer less mechanical support and resistance form compared to the incisal overlap, particularly under functional loading conditions (36). On the other hand, a well-adapted veneer properly designed with proximal “wings” may contribute to periodontal health. Indeed, finite element analysis (FEA) and *in-vitro* studies on extracted teeth have demonstrated that a modified window preparation with convex (“S”-shaped) proximal contours helps preserve the interproximal contact area, minimizes micro-gaps and enhances the aesthetic emergence profile at the gingival papilla. (35, 37, 38) Therefore, all the veneers were decided to prepare with an S-shape proximal contour. Of note, the 6 anterior teeth in this report were prepared following a modified window design, while veneers on the first and second premolars were prepared with partial occlusal coverage to enhance load-bearing capacity and ensure optimal aesthetic integration.

The biomechanics of Angle's Class II Division 2 malocclusion are characterized by anterior protrusive and lateral excursive movements. This occlusal pattern results in the application of substantial functional loads primarily onto the incisal edges of the central incisors and the palatal transitional ridges of the maxillary canines (39, 40). In this study, compensate for the pronounced lingual inclination of the anterior teeth and to provide adequate lip support, the proposed veneers on the four maxillary incisors required a significant thickness of 1.2–2.5 mm at the incisal edge.

Superior clinical outcomes, specifically regarding marginal integrity and bond durability, have been consistently reported with the use of etch-and-rinse adhesive systems (41, 42). In this study, preparation depth was guided by a silicone index to achieve a minimal tooth reduction of 0.3–0.5 mm, ensuring that the preparation margins were located almost within enamel, a paramount factor for achieving a durable and long-lasting bond. Regarding the maxillary central incisors, which presented with rotation and a palatal inclination of the long axis, a heightened risk of dentin exposure following preparation was anticipated. Consequently, following a standardized preparation with an average reduction of 0.3 mm and 0.5–0.8 mm in areas of tooth rotation, iatrogenic dentin exposure was identified at specific sites. An immediate dentin sealing (IDS) protocol was subsequently implemented to prevent postoperative hypersensitivity and to enhance the bond strength of the definitive laminate veneer restorations (43).

Mastering the cementation is another crucial factor for ensuring best performance of LDS veneers. The appropriate luting agent should be chosen following a comprehensive evaluation of factors such as ceramic thickness, adhesive characteristics, precision of marginal fit and the mechanical properties of the cement (44). Dual-cure cements are advantageous in cases where the ceramic material is too thick or too opaque to allow sufficient light transmission for polymerization. However, they have a shorter working time and may lead to failure if not properly cured (45). Previous study has reported that a higher degree of conversion in low viscosity light-cure resin cement materials enhances the mechanical properties, reducing the risk of microleakage and increasing the color stability and durability of the final ceramic veneers (46). In this study, a light-cured luting resin was utilized and the oxygen inhibition layer was further blocked with the glycerine gel application as previously recommended (47). A final high-intensity polymerization cycle was meticulously performed with an LED light source, strictly following the manufacturer's instructions regarding exposure time and working distance.

This report has limitations inherent to a single case study. While clinical and photographic documentation is comprehensive, the absence of systematic periapical radiographs at the 6-year recall (as the patient was asymptomatic) is a limitation for evaluating potential subclinical changes. The long-term performance of the veneers in this specific malocclusion warrants further observation.

## 4 Conclusion

This case report has underscored that the long-term aesthetic and functional success of LDS veneers is contingent upon a comprehensive diagnostic evaluation utilizing the DSD concept, meticulous material selection, appropriate preparation design and precise adhesive cementation protocols.

## Data Availability

The raw data supporting the conclusions of this article will be made available by the authors, without undue reservation.
